# Combining capillary electrophoresis and next-generation sequencing for aptamer selection

**DOI:** 10.1007/s00216-014-8427-y

**Published:** 2015-01-13

**Authors:** Kathryn R. Riley, Jason Gagliano, Jiajie Xiao, Kara Libby, Shingo Saito, Guo Yu, Roger Cubicciotti, Jed Macosko, Christa L. Colyer, Martin Guthold, Keith Bonin

**Affiliations:** 1 Department of Chemistry, Wake Forest University, Winston-Salem, NC 27109 USA; 2 Department of Physics, Wake Forest University, Winston-Salem, NC 27109 USA; 3 NanoMedica LLC, Winston-Salem, NC 27101 USA; 4 Graduate School of Science and Engineering, Saitama University, 255 Shimo-Okubo, Sakura-ku, Saitama, 338-8570 Japan

**Keywords:** Separations, Instrumentation, Capillary electrophoresis, Electrophoresis, Next-generation sequencing, Bioanalytical methods

## Abstract

Next-generation sequencing (NGS) machines can sequence millions of DNA strands in a single run, such as oligonucleotide (oligo) libraries comprising millions to trillions of discrete oligo sequences. Capillary electrophoresis is an attractive technique to select tight binding oligos or “aptamers” because it requires minimal sample volumes (e.g., 100 nL) and offers a solution-phase selection environment through which enrichment of target-binding oligos can be determined quantitatively. We describe here experiments using capillary transient isotachophoresis (*ct*ITP)-based nonequilibrium capillary electrophoresis of equilibrium mixtures (NECEEM) as a method for selecting aptamers from a randomized library containing a known (29mer) thrombin-binding aptamer. Our capillary electrophoresis (CE)-selected samples were sequenced by the Ion Torrent Personal Genome Machine (PGM) and analyzed for selection efficiency. We show that a single round of CE selection can enrich a randomer synthetic DNA oligo mixture for thrombin-binding activity from 0.4 % aptamer content before selection to >15 % aptamer content.

**Electronic supplementary material:**

The online version of this article (doi:10.1007/s00216-014-8427-y) contains supplementary material, which is available to authorized users.

## Introduction

Here, our purpose is to demonstrate how to 1) optimize aptamer selection using capillary transient isotachophoresis (*ct*ITP)-based nonequilibrium capillary electrophoresis of equilibrium mixtures (NECEEM) and to 2) quantify and analyze aptamer selection using the Ion Torrent Personal Genome Machine (PGM). We demonstrated our combination of capillary electrophoresis (CE) and next-generation sequencing (NGS) using the 29-nucleotide thrombin aptamer “29mer” that binds thrombin protein. This new combination of methodologies can be extended to yet undiscovered aptamers.

CE is a powerful separation technique. Recently, novel kinetic CE (KCE) techniques, such as NECEEM, have been used to select aptamers from randomer libraries [1, 2], improving over traditional systematic evolution of ligands by exponential enrichment (SELEX) [3, 4] in speed and sample conservation.

This work uses NECEEM [5, 6] to separate aptamer-target complexes from oligo libraries that contain one target-binding aptamer. In NECEEM, the target and oligo library interact in solution prior to electrophoretic separation. We have recently demonstrated improvements on NECEEM by developing new fluorescent derivatization techniques for labeling of oligonucleotide libraries and also by using capillary transient isotachophoresis, *ct*ITP, to improve resolution of aptamer-target complexes from the bulk library [7]. In *ct*ITP, leading and terminal ions (which have mobilities greater than or less than the analyte, respectively) are used to first focus the analyte band as in traditional isotachophoresis, and then as the ions eventually de-stack, the analytes are separated as in traditional capillary zone electrophoresis (CZE) [8]. In this work, a leading ion was added to the sample buffer and a terminal ion was added to the separation buffer.

Five conditions should be met to optimally combine CE and NGS for aptamer selection. First, oligo libraries must be prepared with DNA “handles” (flanking adaptor sequences for amplification and sequencing) that do not interfere with aptamer folding. Second, the reaction mixture applied to the capillary must contain purified target and at least one oligo species (aptamer) with sufficiently high target-binding affinity to allow separation of the aptamer-target complex from nonbinding species. Third, the separated aptamer-target complex should be detectable, which we addressed through improved separation and labeling methods [7] (see also Experimental section) to enable detection of 10 femtograms (fg) of single-stranded DNA. Fourth, each CE collected fraction requires sufficient DNA to avoid excessive cycles of PCR, which can result in amplification artifacts such as long DNA concatemers (data not shown). Fifth, all NGS systems require sophisticated data analysis. The PGM is known to make base-calling errors for long homopolymer sequences [9], a limitation we addressed through data filtering.

With these conditions in mind, we combined CE and NGS to select the 29mer thrombin aptamer spiked into a library with ∼10^15^ unique oligonucleotides. Our proof-of-concept study established the following: (i) *ct*ITP aptamer selection and enrichment can be quantified by NGS, (ii) the aptamer fraction can be enriched >40-fold following a single round of *ct*ITP selection, and (iii) any potential secondary structure of the quadruplex-forming thrombin aptamers does not seriously impede Ion Torrent NGS sequencing. Additionally, our work established that NGS sequencing by the Ion Torrent PGM is reproducible from run to run and is independent of sequencing chip size, automated emulsion PCR in the Ion One Touch system, and enrichment. We have also established that the addition of co-ions (K^+^, Na^+^, and Mg^2+^) decreased our ability to select the thrombin aptamer against a randomer library due to a decrease in complex formation as well as a deterioration in separation resolution as seen in other labs [10].

## Experimental

**Fig. 1:**
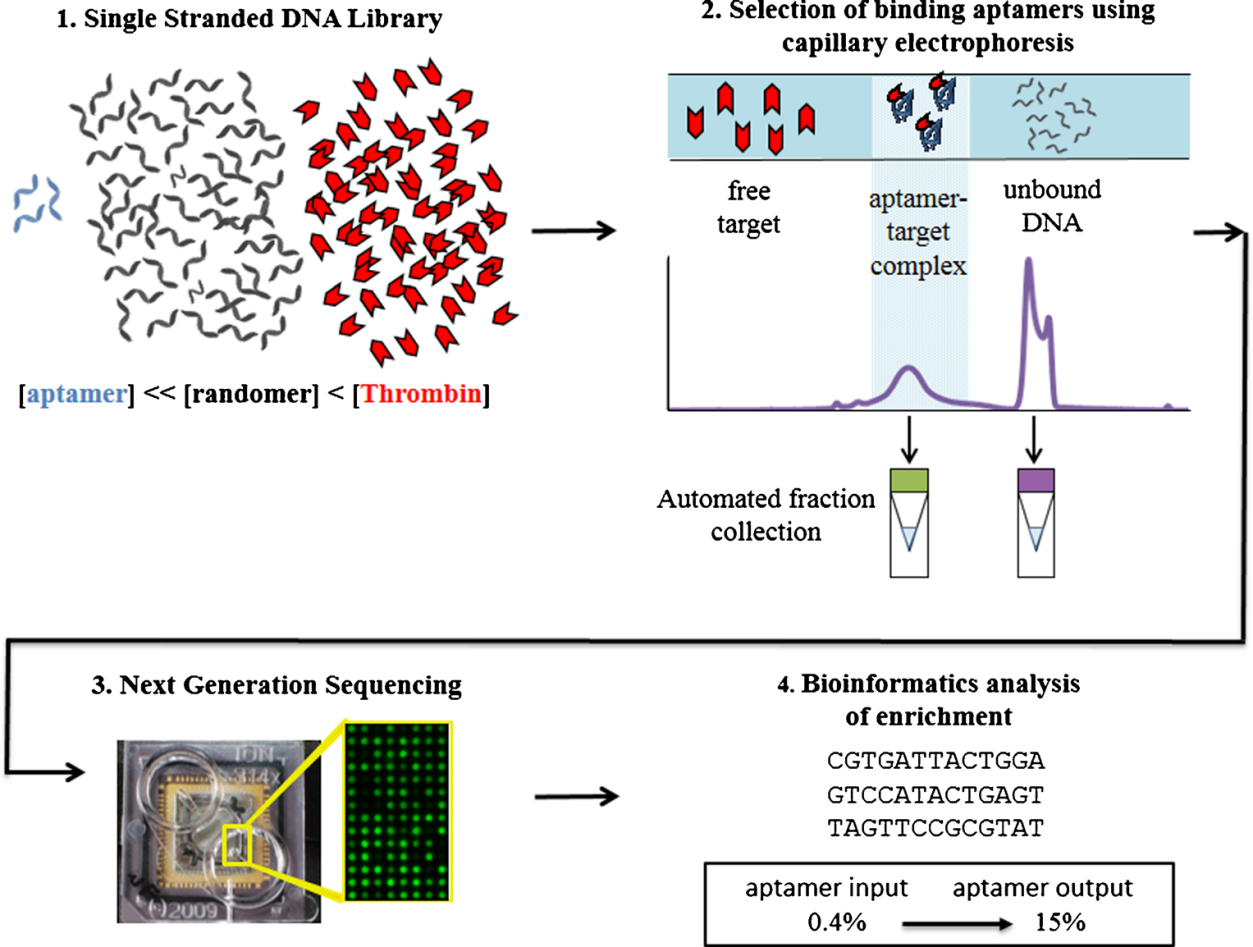
Flow chart for CE selection study. *Step 1*: A mixture of thrombin aptamer, randomer, and thrombin protein is prepared, with aptamer concentration far lower than either randomer or protein. *Step 2:* The mixture is injected onto the capillary, separated, and both the aptamer-target complex peak and the unbound DNA peak are collected. *Step 3:* Fractions are loaded onto a sequencing chip (*inset*: 30 μm × 50 μm fluorescent image of Ion Torrent’s bead-based sequencing platform). *Step 4:* Bioinformatics analysis of CE selection, which shows an average increase in aptamer content from 0.4 to 15 % (see Electronic supplementary material (ESM) Table S1)

We present a schematic of the experimental workflow in Fig. 1. The basic steps are to start by mixing a ssDNA aptamer library (spiked 29mer randomer; for details about the 29mer randomer, see the supplement) with the target (thrombin) and then separate the aptamer-target complex using CE. Next, sequence the pre-injected “original” aptamer-target “complex” and unbound-“free” DNA samples with subsequent bioinformatics analysis.

### Chemicals and reagents

We purchased tris (hydroxymethyl) amino methane (Tris) and glycine (Gly) from Sigma (St. Louis, MO); SYBR Gold, Dynabeads MyOne Streptavidin C1, Ion PGM Template OT2 200 Kit, Ion Sphere Quality Control Kit, Ion PGM Sequencing 200 Kit v2, Ion 314 Chip Kit v2, and Ion 316 Chip Kit from Life Technologies (Carlsbad, CA); human *α*-thrombin from Enzyme Research Laboratories (South Bend, IN), DNA samples and Ion Torrent primers (including the ∼10^15^ member randomer library, see Supplementary Materials–Note 1) from IDT (Coralville, IA); NEBNext Fast Library Prep Set for Ion Torrent kit from New England Biolabs (Ipswich, MA); Agencourt AMpure XP from Beckman Coulter (Brea, CA); Ethyl Alcohol from Fisher Scientific (Pittsburgh, PA); and DNA Reagent kit from Agilent Technologies (Santa Clara, CA).

### Capillary electrophoresis

We prepared “Tris–HCl sample buffer” (pH 8.2, 50 mM Tris) and “Tris–Gly separation buffer” (pH 8.2, 31 mM Tris and 500 mM Gly). The Tris–HCl sample buffer was the leading electrolyte (chloride anion was the leading ion), and the Tris–Gly separation buffer was the terminal electrolyte (glycine anion was the terminal ion). We filtered all buffers (0.20 μm filter) prior to use. We labeled DNA “on-column” with SYBR gold/Tris–Gly separation buffer (1:100,000). We prepared stock DNA and thrombin samples (2 μM) in Tris–HCl sample buffer and heated the stock DNA for 5 min at 95 °C and allowed it to cool slowly to room temperature. We prepared working samples by diluting stock solutions to the appropriate concentration with Tris–HCl sample buffer. We incubated DNA samples with thrombin for 20 min at 4 °C prior to injection. Incubation of samples at room temperature (25 °C) may be beneficial for maximum complex formation [11]. However, under our experimental conditions, we observed no differences in the amount of complex formed as a function of incubation temperature (data not shown). Thus, for consistency, we refrigerated all samples at 4 °C until analysis.

We performed all CE studies on a Beckman Coulter P/ACE MDQ CE System with laser-induced fluorescence (LIF) detection (Ar-ion laser excitation at 488 nm, 520 nm long pass, and 488 nm notch filters). We used 32 KARAT software for CE control and analysis. The capillary was an uncoated fused-silica capillary (Polymicro Technologies, Phoenix, Arizona, 60.2 cm total length, 50.0 cm effective length, from inlet to detector, and 75 μm internal diameter). We filled the capillary and the inlet and outlet vials, with Tris–Gly separation buffer. The collection vial contained 10 μL of 400 nM thrombin protein in Tris–HCl sample buffer. We injected samples onto the capillary for 4 s at 5 psi, separated at 18.0 kV, and collected at 10 kV.

After testing 100, 200, 400, 600, 800, and 1000 nM thrombin against 200 nM aptamer, we concluded that 400 nM thrombin was optimal for detection and resolution. We prepared the spiked randomer library by mixing 29mer thrombin aptamer (2 nM) and randomer (200 nM) in Tris–HCl sample buffer and incubated it for 30 min with 400 nM thrombin. Each day, we ran this mixture in triplicate to identify the collection window (see ESM Fig. S1). We adjusted the collection window as needed to account for day-to-day variations in migration time. We collected aptamer-thrombin complex peak and unbound DNA peak in 16 successive runs over 4 h. We gently vortexed each volume collected to homogenize the sample and then we stored the samples at 4 °C until NGS analysis.

Each day, we injected 0.5, 2.5, 10, and 25 nM aptamer standards prepared in Tris–HCl sample buffer and containing 400 nM thrombin protein onto the capillary (*n* = 3) and constructed a calibration plot based on peak area. With this calibration curve, we determined the concentrations of the collected and re-injected samples (*n* = 3).

Each injection of 180 nL of 200 nM randomer DNA contained 2.16 × 10^10^ oligos (i.e., 3.46 × 10^11^ for 16 runs), which is 1/3000th of the ∼10^15^ member randomer library.

### NGS preparation, measurement, and analysis

We amplified the samples (“original,” “complex,” and “free”) using NEBNext Fast DNA Library Prep Set reagents with 1 nM DNA PCR reaction mix, 10 μM each of IDT Ion Torrent A primer, and truncated P1 primer (for PCR conditions, see Supplementary Materials–Note 2). We cleaned up samples using Agencourt AMPure XP (protocol 000387v001), and we analyzed the samples for size and concentration on an Agilent 2100 Bioanalyzer.

We performed bead preparation and sequencing according to the Ion PGM user guides, Catalog Number 4480974, Publication Number MAN0007220, Revision 4.0 (using 10 pM DNA in the OneTouch2) and Catalog Number 4482006, Publication Number, MAN0007273, Revision 1.0 respectively.

We used NGS to characterize the efficiency of CE selection of the 29mer aptamer based on the overrepresentation of its sequence in the complex fraction relative to the original prepared library sample. Since direct pattern search is affected by sequencing errors, e.g., insertions/deletions, we implemented a Smith-Waterman algorithm using MatLab (Natick, MA) running on the DEAC computer cluster at Wake Forest University. We also analyzed the sequence frequency using the extract and count function in the CLC Genomics Workbench (CLC bio, Aarhus, Denmark). We trimmed the first 15 nucleotides of all the reads to remove the common spacer sequence present in the library between the 29mer and the Ion Torrent Primer A.

## Results and discussion

Previous work optimized the *ct*ITP method by using SYBR Gold as an on-column, noncovalent label for single-stranded DNA (see ESM Fig. S2) and obtained *K*_d_ = 124 ± 6 nM for aptamer-thrombin dissociation [7], which is consistent with published *K*_d_ values for the 29mer [11, 12]. This demonstrated that neither on-column labeling nor *ct*ITP focusing interfered with aptamer-thrombin binding and that the Ion Torrent adapters did not unduly affect aptamer affinity.

The 29mer folds into a G-quadruplex/partial duplex structure [13]. One study found that K^+^ ions increased the circular dichroism signal of the aptamer at the wavelength associated with G-quadruplex structure [14]. However, other CE analyses have concluded that K^+^ either did not increase complex formation [10, 11] or that K^+^ or Mg^2+^ decreased complex formation [10]. Given these mixed results, we studied the effect that co-ions have on aptamer binding. We injected 200 nM 29mer and 200 nM thrombin onto the capillary either in the absence or presence of 10 mM Mg^2+^, Na^+^, or K^+^ (see ESM Fig. S3). Our addition of a co-ion resulted in decreased fluorescence signal and shifts in migration time. Na^+^ and K^+^ had the added disadvantage of decreased resolution between the complex and free DNA bands, which is consistent with a CE study reporting fluorescence decreases in the presence of K^+^ and Mg^2+^ [10]. Moreover, all co-ions resulted in decreased resolution (ESM Fig. S3). Therefore, we conducted our studies in the absence of any metal ions.

**Fig. 2:**
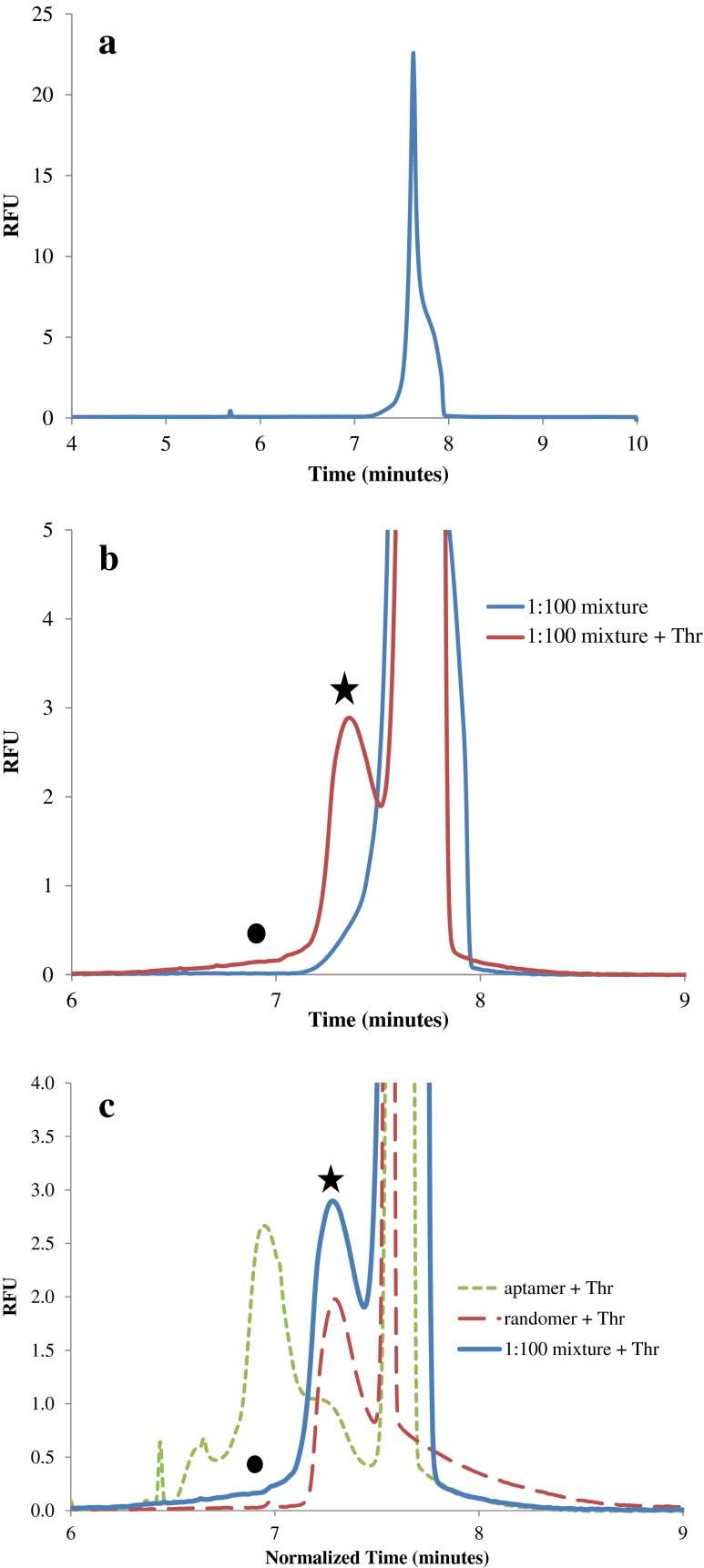
Electropherograms from CE aptamer selection. **a** A single peak is observed for an injection of the 1:100 DNA mixture (2 nM aptamer, 200 nM randomer). **b** When thrombin (400 nM) is added to the 1:100 mixture, two new signals are observed as indicated by the *circle* and *star*. The signal indicated by the circle in **b** has the same migration time as the specific aptamer-thrombin complex peak (*left green peak* in **c**), while the peak indicated by the *star* in **b** has the same migration time as the nonspecific “complex” peak observed between the randomer library and thrombin (*left red peak* in **c**)

Injecting a 1:100 mixture of 29mer/randomer onto the capillary resulted in a single peak at 7.6 min in the electropherogram (Fig. 2a). When thrombin was added, we observed increased intensity from 6.3 to 7.2 min (circles, Figs. 2b, c) and a shoulder at 7.2 min (stars, Figs. 2b, c). Control experiments with the randomer only plus thrombin revealed a shoulder at 7.2 min (Fig. 2c, red trace) and with a sample of the aptamer only plus thrombin a peak occurred at 6.9 min (Fig. 2c, green trace). This peak, corresponding to the complex between 29mer and thrombin, was used to establish the collection windows (as illustrated in ESM Fig. S1) and demonstrates the resolving power of CE to distinguish specific (29mer/protein; circle, Fig. 2c, blue trace) from non-specific (random DNA/protein; star, Fig. 2c, blue trace) interactions.

A histogram of matched bases before and after CE selection reveals that the 29mer was well selected (see Fig. S4). We used three different groups of penalty scores (see Supplementary Materials–Note 3) in the Smith-Waterman algorithm (local alignment gave similar results, Figs. S5-6). We chose 24 matching bases as the threshold to judge whether a measured sequence corresponds to the 29mer. However, except for the first run in Fig. S7 (Set 1–complex), the ratio of 29mer-to-28mer sequences generally exceeded 90 %.

Our figure of merit is the enrichment factor, *f*_E_, defined as:

1 $$ {f}_E=\frac{\mathrm{fraction}\ \mathrm{of}\ \mathrm{aptamer}\ \mathrm{sequences}\ \mathrm{in}\ \mathrm{complex}\ \mathrm{peak}}{\mathrm{fraction}\ \mathrm{of}\ \mathrm{aptamer}\ \mathrm{sequences}\ \mathrm{in}\ \mathrm{original}\ \mathrm{in}\mathrm{jected}\ \mathrm{signal}}. $$

The numerator of Eq. (1) represents the number of aptamer sequences identified in the collected complex sample (region I in ESM Fig. S1) divided by the total number of sequencing reads (aptamer and non-aptamer). The denominator is the number of aptamer sequences identified in the original library sample (the 1:100 aptamer/randomer mixture) divided by the total number of sequencing reads. This enrichment factor is given in Table S1 for all of our experimental runs (refer also to Fig. 2 and ESM S1). Each CE run exhibited enrichment, although the magnitude of the enrichment varied 6- to 93-fold with an average enrichment of 38.

The ability of CE to select target-binding candidate molecules makes it a potentially powerful tool to discover aptamers and other drug candidates, as can be seen in Fig. 2. The complex/free ratio (red trace, Fig. 2b, circle vs. right-most peak and starred peak) averages 0.88 %, close to the original 1 % aptamer concentration. However, with 2 nM aptamer, 400 nM thrombin, and *K*_d_ = 124 nM, only 76 % of the aptamers should be bound, so the complex peak should be ∼¾ aptamer and ∼¼ other DNA. Thus the fraction of complex in the complex peak ~ 0.88 % is close to the expected value of 0.76 % based on our concentrations and the binding constant.

Averaging column 2 in Table S1(ESM) yields 0.39 % aptamer content in the starting library sample, which is an efficiency of 39 % (since we measured 0.39 % instead of the expected 1 %), so, in a library with 1000 copies of each member, 390 strands of a strong binding (*K*_d_ < <124 nM) aptamer will be amplified for sequencing. If the CE enhancement factor is 38, only 1000/38 = 26 strands of any other library member would be amplified for sequencing in the collected complex sample. This means the probability for proper identification of a strong binder in a collected CE fraction via NGS is extremely high, virtually 100 %. Such an extensive overrepresentation of one sequence among many demonstrates the robustness of this combined technique in finding new aptamers.

The two sample runs in “Set 3” (ESM Table S1) independently went through the process of emulsion PCR on the Ion Torrent One Touch, enrichment of templated beads, and loading onto different sized chips. Set 3a was loaded onto a 316 chip (six million wells), and Set 3b was loaded onto a 314 chip (one million wells). The results in Table S1 (ESM) show that there was no bias introduced by the different chips or sample prep. Hence, the One Touch, enrichment module, and PGM are a reliable means to investigate an aptamer DNA library for drug discovery.

## Conclusions

In summary, we have described a combined CE and NGS system that can be used for aptamer-based molecular/drug discovery. This paper reports on the efficacy and efficiency of using CE as a library enrichment tool for aptamer selection using the NGS system. Our experiments demonstrate, for the first time, that capillary transient isotachophoresis (*ct*ITP)-based nonequilibrium capillary electrophoresis of equilibrium mixtures (NECEEM) is an excellent CE modality for our selection purposes. We showed that a single round of CE selection can, on average, enrich an aptamer-randomer mixture from 0.4 to >15 %. This level of enrichment will enable the NGS platform to resolve, with very high probability, the best aptamers for targets of interest in a variety of applications, including the discovery of new drug candidates and diagnostic reagents. The small sample volume requirement and high degree of enrichment make CE an ideal selection technique to aid NGS-based aptamer and drug discovery. Furthermore, based on a previous application [15] of NGS to determine aptamer-protein binding constants, we envision a novel “reversed-order” aptamer discovery approach, where an oligonucleotide library would first be sequenced by next-generation sequencing followed by exposure of the chip to a labeled target for identification of target-binding sequences. In this approach, CE could serve as a *pre*selection tool to reduce the size of the candidate library followed by on-chip NGS sequence identification and selection. In either case—traditional CE-SELEX followed by NGS analysis of the obtained sequences or a novel reversed-order approach in which CE is used as a preselection technique followed by on-chip aptamer selection—CE coupled with Ion Torrent NGS represents a powerful combination for aptamer selection.

## Electronic supplementary material

Below is the link to the electronic supplementary material.

ESM 1 (PDF 545 kb)

## Abbreviations

CE: Capillary electrophoresis
*ct*ITP: Capillary transient isotachophoresis
ECEEM: Equilibrium capillary electrophoresis of equilibrium mixtures
emPCR: Emulsion polymerase chain reaction
KCE: Kinetic capillary electrophoresis
NECEEM: Nonequilibrium capillary electrophoresis of equilibrium mixtures
NGS: Next-generation sequencing
PGM: Personal Genome Machine
SELEX: Systematic evolution of ligands by exponential enrichment
