# Supplementary material for: Combining capillary electrophoresis and next-generation sequencing for aptamer selection
Source: Anal Bioanal Chem. 2015 Jan 13;407(6):1527–32. doi: 10.1007/s00216-014-8427-y (PMC4329186; doi:10.1007/s00216-014-8427-y)
Supplement: Supplementary file 1 — (PDF 545 kb) [file 216_2014_8427_MOESM1_ESM.pdf]

Analytical and Bioanalytical Chemistry

Electronic Supplementary Material

Combining capillary electrophoresis and next-generation sequencing for aptamer selection

Kathryn R. Riley, Jason Gagliano, Jiajie Xiao, Kara Libby, Shingo Saito, Guo Yu, Roger Cubicciotti, Jed Macosko, Christa L. Colyer, Martin Guthold, Keith Bonin

Note 1. DNA samples and Ion Torrent primers were based on a 29mer randomized at the central 25 positions with Ion Torrent primer handles and a spacer sequence (5'- CCT CTC TAT GGG CAG TCG GTG AT**A GNN NNN NNN NNN NNN NNN NNN NNN NNC T**TA AGG AGT TCA ATA TCT GAG TCG GAG ACA CGC AGG GAT GAG ATG G-3', 97 bases), 29mer thrombin aptamer with Ion Torrent primer handles and a spacer sequence (5'- CCT CTC TAT GGG CAG TCG GTG AT**A GTC CGT GGT AGG GCA GGT TGG GGT GAC T**TA AGG AGT TCA ATA TCT GAG TCG GAG ACA CGC AGG GAT GAG ATG G-3', 97 bases), Ion Torrent Primer A (5'-CCA TCT CAT CCC TGC GTG TCT CCG ACT CAG-3'), and unlabeled Ion Torrent truncated P1 primer (5'-CCT CTC TAT GGG CAG TCG GTG AT-3').

Note 2. The PCR conditions for the original and free DNA samples included denaturation (30 sec, 98ºC), 5 cycles of denaturation (10 sec, 98ºC), annealing (30 sec, 58ºC), extension (30 sec, 72ºC), and a final extension (5 min, 72ºC). PCR conditions for the collected complex sample included denaturation (30 sec, 98ºC), 10 cycles of denaturation (10 sec, 98ºC), annealing (30 sec, 58ºC), extension (30 sec, 72ºC), and a final extension (5 min, 72ºC).

Note 3. Bioinformatics - three different groups of penalty scores were used in the Smith-Waterman algorithm (Match = 1, Mismatch = -1/3, Indel = -1, named as alignment 1 below), (Match = 1, Mismatch = -2, Indel = -1, named as alignment 2) and (Match = 5, Mismatch = -4, Indel = -8, named as alignment 3). See Figs S5 and S6.

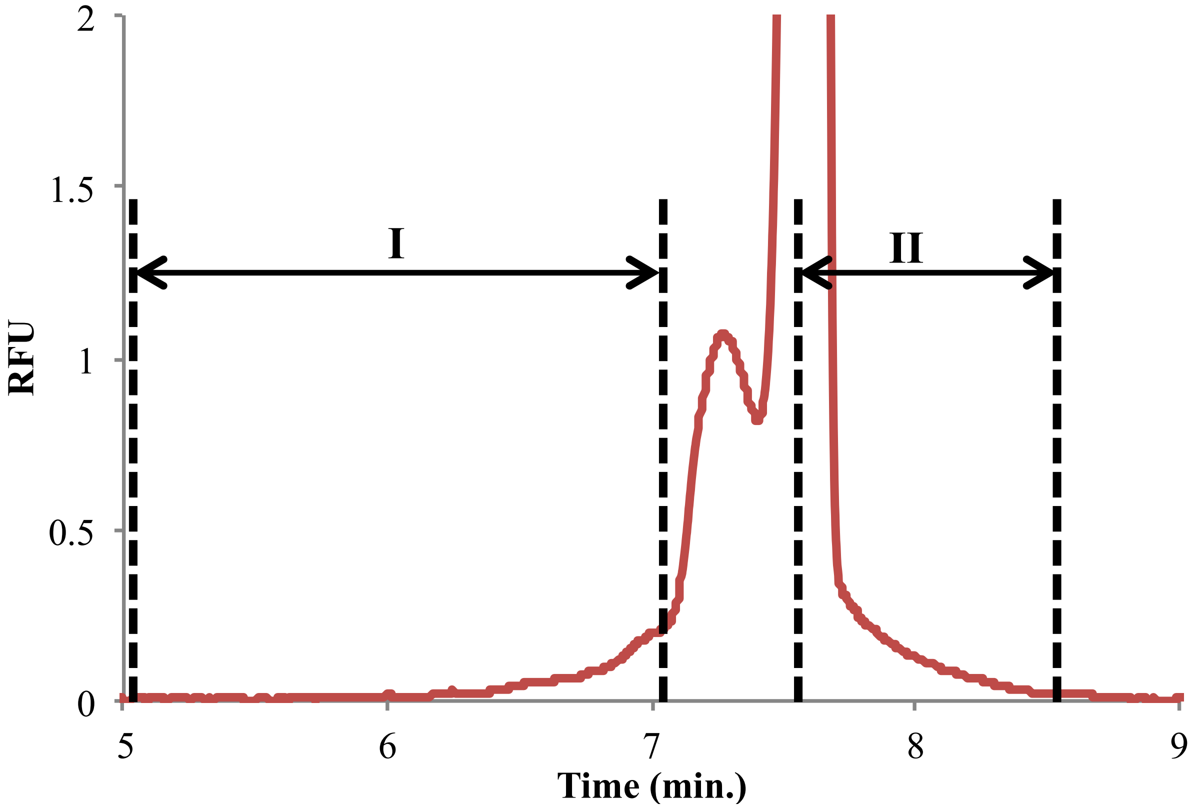

Fig. S1. Collection windows for the thrombin aptamer-thrombin protein complex peak (I) and for the free (unbound) DNA (II).

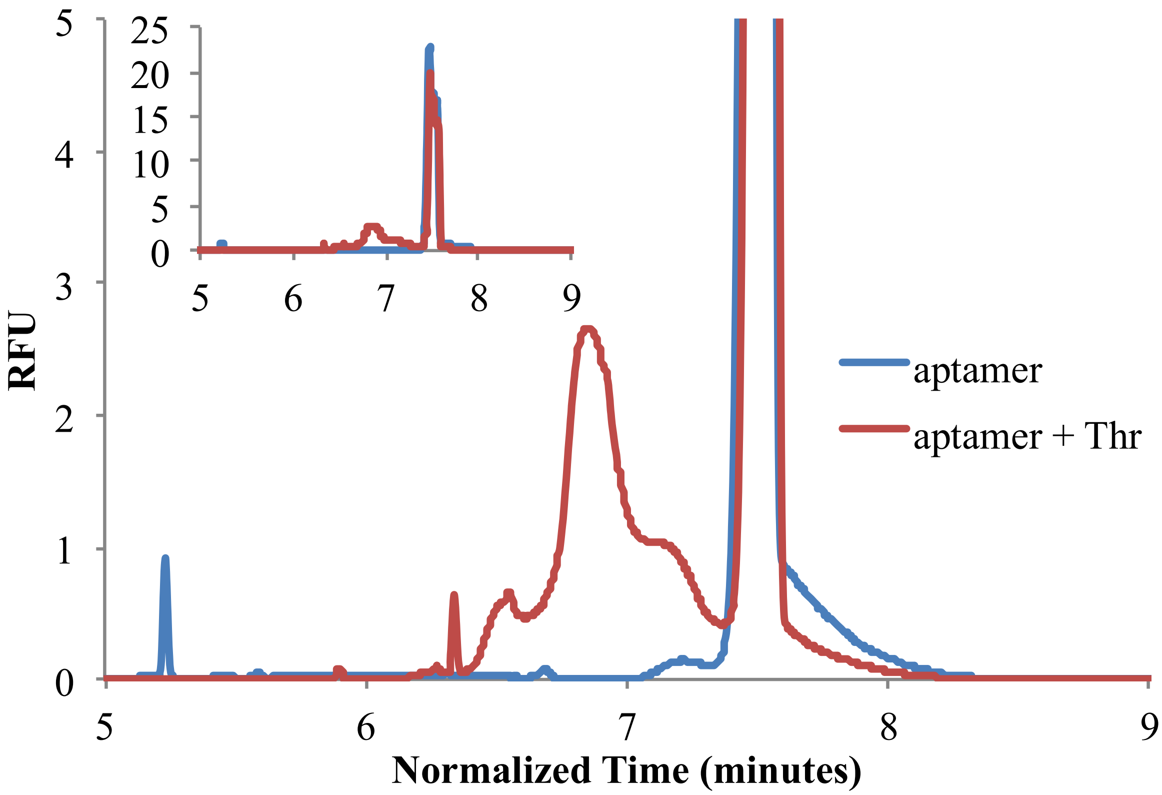

Fig. S2. Electropherograms of the thrombin aptamer alone (200 nM, blue trace) and in the presence of 400 nM thrombin (red trace). The formation of a peak at ~6.8 minutes was indicative of the formation of thrombin aptamer-thrombin protein complex. The subsequent decrease in the unbound DNA peak at 7.5 minutes in the red trace relative to the blue trace (see inset) further confirmed complex formation.

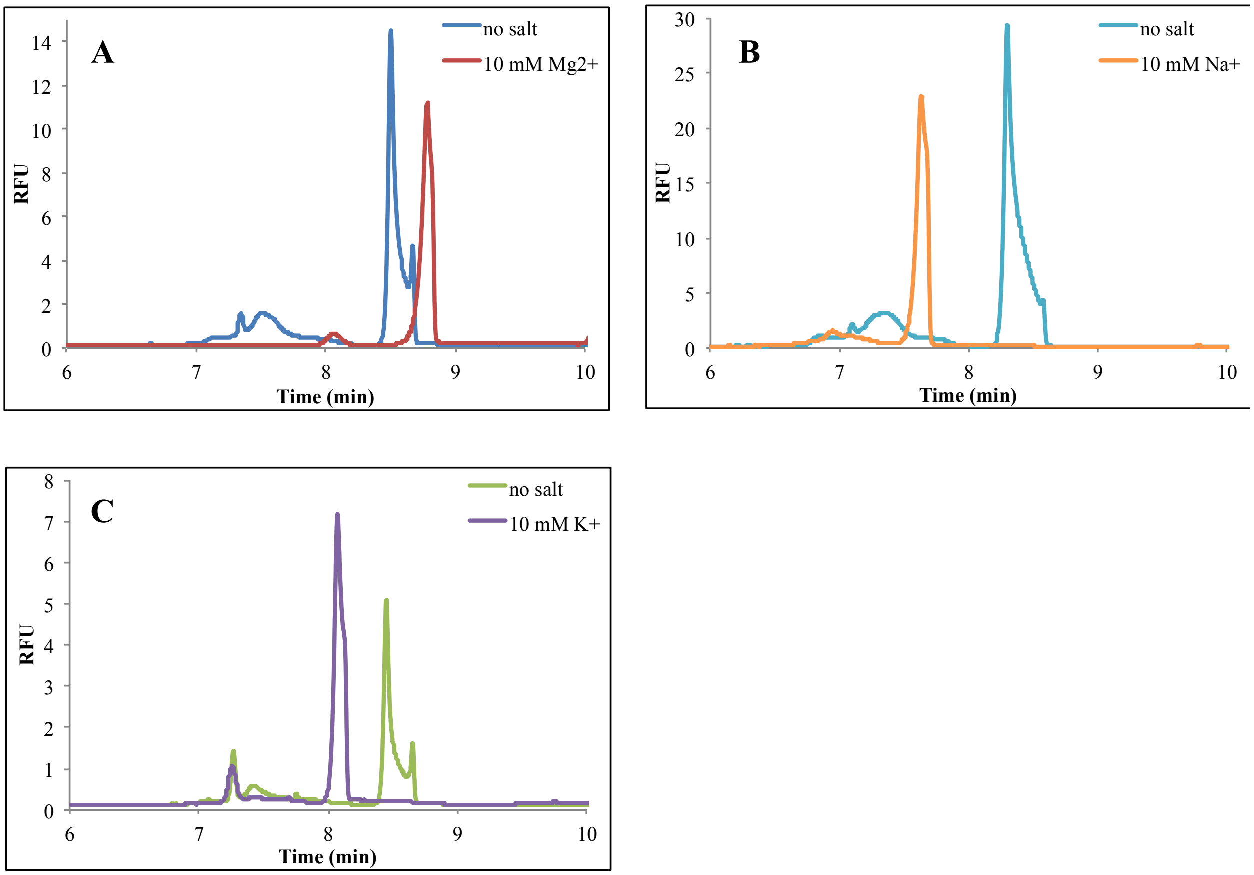

Fig. S3. Electropherograms demonstrating the addition of a co-ion to a sample of the thrombin aptamer (200 nM) plus 200 nM thrombin and the co-ion's effect on the *ct*ITP separation. In all cases electropherograms were recorded without salt and again with the addition of 10 mM (A) Mg^{2+}, (B) Na^{+}, and (C) K^{+}. The addition of each co-ion was accompanied by a decrease in fluorescence signal and a shift in migration time. The addition of Na^{+} and K^{+} resulted in a decrease in resolution of the bound and unbound forms of the aptamer. These experiments were conducted using a 1:500,000 dilution of SYBR Gold, which resulted in a difference in electroosmotic flow (EOF) and consequently a difference in migration time of the bound and unbound forms of the aptamer relative to other experiments presented in this work.

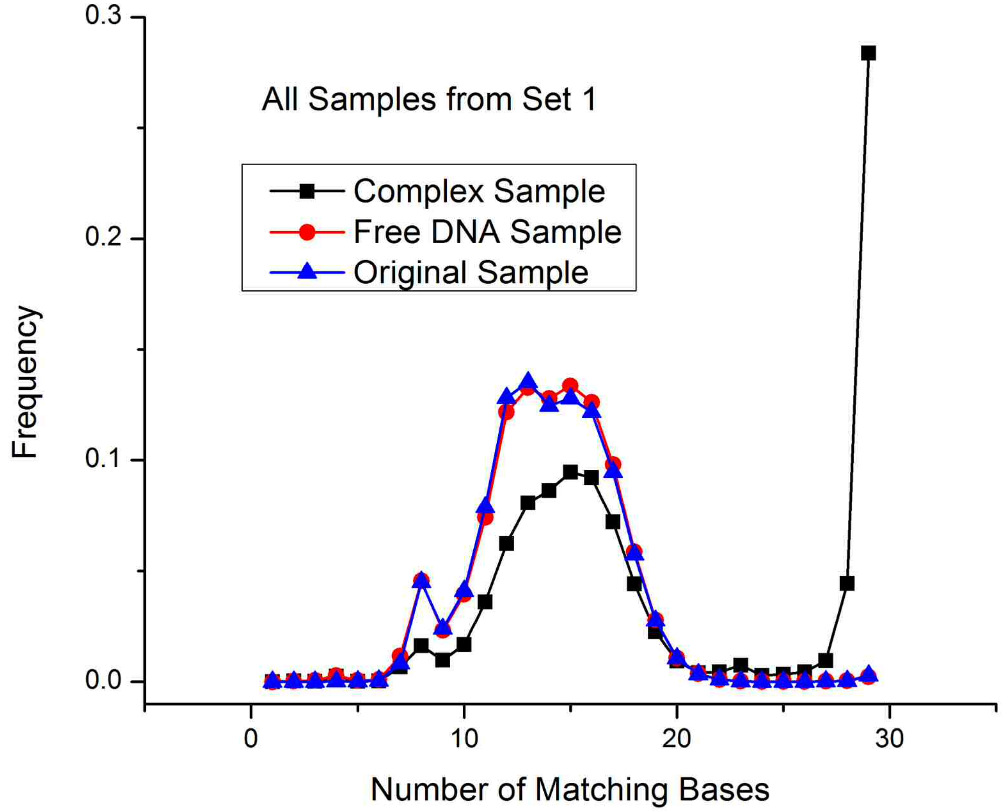

Fig. S4: A typical matching histogram for the sequences from the complex, the free DNA and the original samples. The percentage of the sequences with at least 27 matching bases to the 29mer aptamer is significantly higher in the complex sample in comparison with the tiny peak for the 29mer aptamer in the original and the free DNA sample. The middle 'peaks' represent the 29mer-randomers, which can have around 15 bases matching the actual aptamer under this alignment. The small 'peaks' at 8 bases correspond to the spacer sequence. Samples from selection of set 3 and sequencing run 1 and sequence analysis results from the alignment 1 were used as an example.

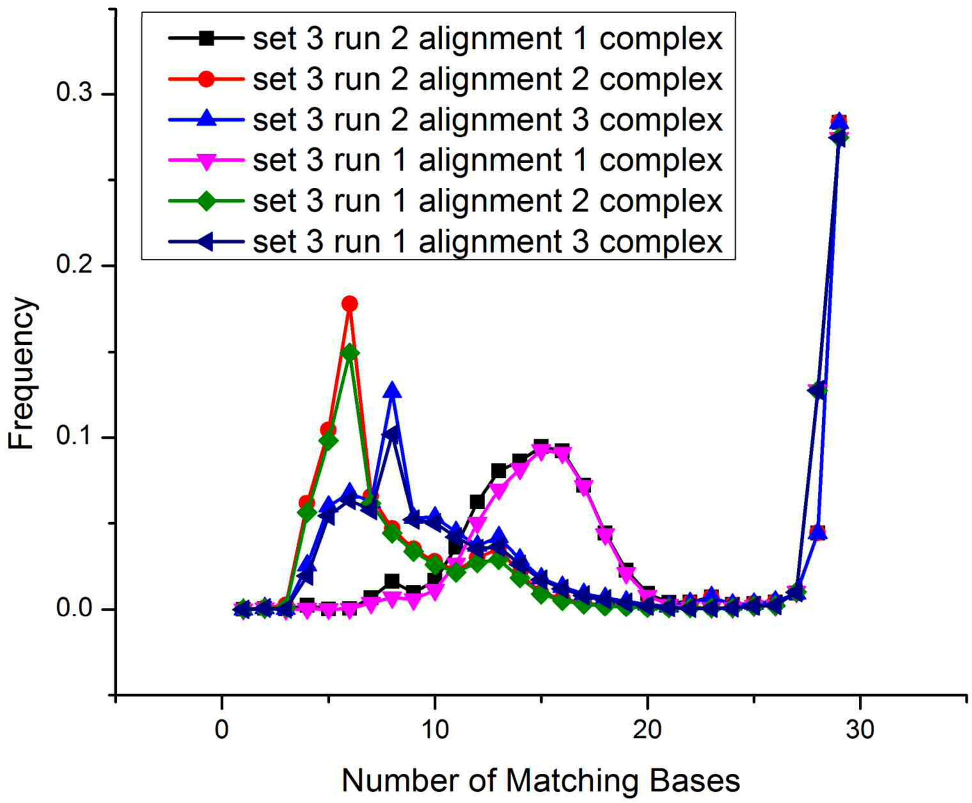

Fig. S5: Matching histograms for three groups of penalty scores for alignments on two sequencing runs – Set 3, runs 1 & 2. Different scores for indels result in different best alignments. The peak corresponding to the maximum number of matching bases of the randomers significantly shifts with the different indel scoring. However, the differing indel penalty scores do not significantly change the correct maximum (or the distribution) of the 29-mer-aptamer (matches above 20 bases).

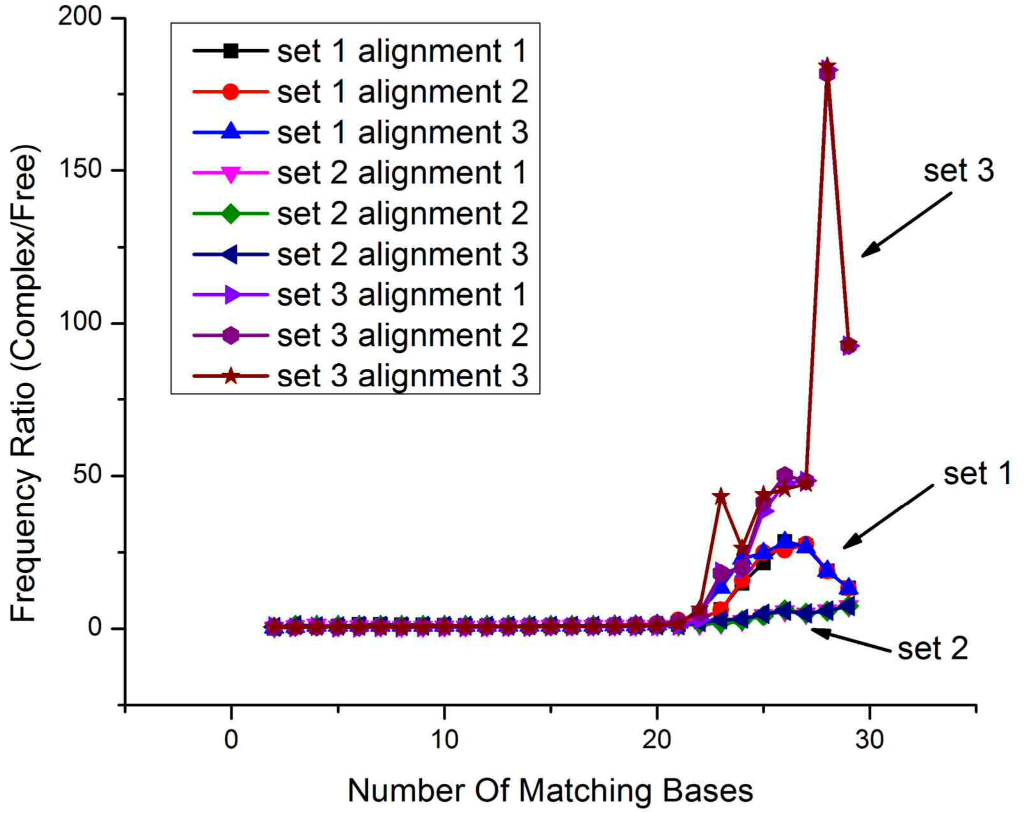

Fig. S6: Ratio of enrichment factors of the complex peak to the free DNA peak. Note that this ratio is fairly flat for low numbers of bases and only becomes significantly greater than 1 close to 23-24 bases.

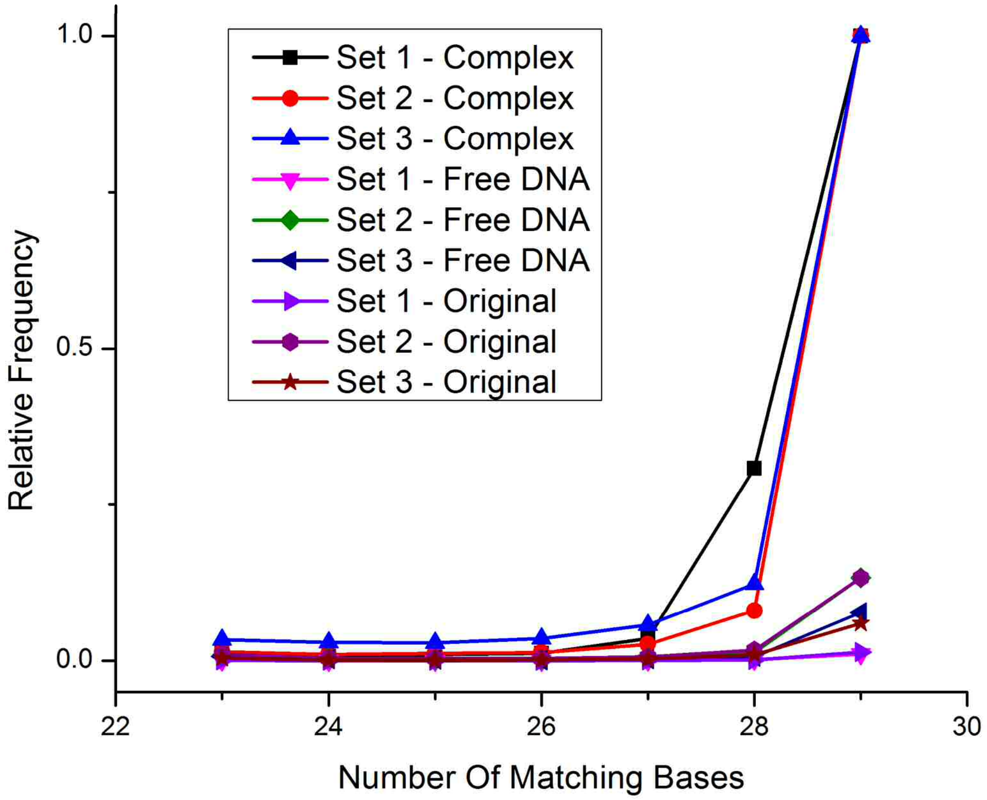

Fig. S7: The relative frequencies of sequences with 23-29 matching bases were obtained by normalizing the frequencies by the maximum frequency of each set. The peaks for the existence of 29mer aptamer in the complex sample are clearly separated from the one in the free DNA and the original sample, no matter which set of selections the data is calculated from. Deviations due to differing alignments are smaller than the size of the markers.

Table S1: Aptamer enrichment by CE-based selection.

Replicate*	% Input Aptamer (CLC)	% Output, bound and free (CLC)	f_E (Output/ Input), bound and free (CLC)	f_E(avg) (Average Enrichment), bound and free (CLC)
set 1 (1 run)	0.37 (0.27)	6.08 (5.12)	16.6 (19.4)	16.6 (19.4)
		0.44 (0.36)	1.2 (1.4)	1.2 (1.4)
set 2 (2 runs)	0.38 (0.30)	3.01(2.75)	7.8 (9.2)	
		0.42 (0.39)	1.1 (1.3)	6.9 (7.7)
	0.45 (0.42)	2.67 (2.45)	5.9 (6.1)	1.0 (1.1)
		0.38 (0.34)	0.8 (0.81)	
set 3 (2 runs)	0.57 (0.49)	41.76 (26.8)	73.4 (54.7)	
		0.44 (0.34)	0.8 (0.82)	83.0 (72.5)
	0.38 (0.31)	34.85 (28.0)	92.5 (90.3)	0.8 (0.78)
		0.34 (0.23)	0.9 (0.74)	

*CE selection was performed a total of three times resulting in 3 "sets" of complex, free, and original DNA samples. Set 1 was sequenced on a 314 chip. Set 2 was subjected to emPCR (emulsion polymerase chain reaction) to create the bead library, and half of the bead library was sequenced (run 1), while the 2nd half of the library was sequenced two weeks later (run 2). Set 3 samples were split in half and subjected to emPCR separately to create two separate bead libraries, one of which was loaded on a 316 chip (run 1) and the other of which was loaded on a 314 chip (run 2). Values listed in parentheses are based on analyses carried out using the CLC Genomics Workbench.

Table S2: CE-based selection and NGS workflow system efficiency.

Replicate[a]	% input aptamer from sequencing analysis (CLC)	% input aptamer from CE area analysis
Set 1 (1 run)	0.37 (0.27)	0.68
		0.73
Set 2 (2 runs)	0.38 (0.30)	1.10
		1.05
	0.45 (0.42)	1.08
		1.35
Set 3 (2 runs)	0.57 (0.49)	0.75
		0.70
	0.38 (0.31)	0.71
		0.68

[a]Replicates as defined in the footer of Table S1.
